# The effective bioengineering method of implantation decellularized renal extracellular matrix scaffolds

**DOI:** 10.18632/oncotarget.5304

**Published:** 2015-09-21

**Authors:** Yong Guan, Shuangde Liu, Chao Sun, Guanghui Cheng, Feng Kong, Yun Luan, Xiaoshuai Xie, Shengtian Zhao, Denglu Zhang, Jue Wang, Kailin Li, Yuqiang Liu

**Affiliations:** ^1^ The Second Hospital, Shandong University, Department of Urology, Shandong, China; ^2^ The Second Hospital, Shandong University, Department of Kidney Transplantation, Shandong, China; ^3^ The Second Hospital, Shandong University, Department of Central Research Lab

**Keywords:** scaffold, tissue engineering, kidney regeneration, decellularization, recellularization

## Abstract

End stage renal disease (ESRD) is a progressive loss of kidney function with a high rate of morbidity and mortality. Transplantable organs are hard to come by and hold a high risk of recipient immune rejection. We intended to establish a more effective and faster method to decellularize and recellularize the kidney scaffold for transplant and regeneration. We successfully produced renal scaffolds by decellularizing rat kidneys with 0.5% sodium dodecyl sulfate (SDS), while still preserving the extracellular matrix (ECM) 3D architecture, an intact vascular tree and biochemical components. We recellularized the kidney scaffolds with mouse embryonic stem (ES) cells that then populated and proliferated within the glomerular, vascular, and tubular structures. After *in vivo* implantation, these recellularized scaffolds were easily reperfused, tolerated blood pressure and produced urine with no blood leakage. Our methods can successfully decellularize and recellularize rat kidneys to produce functional renal ECM scaffolds. These scaffolds maintain their basic components, retain intact vasculature and show promise for kidney regeneration.

## INTRODUCTION

Chronic kidney disease is a major cause of mortality and morbidity worldwide, affecting about 8% to 16% of the global adult population [1]. Currently, the only available treatments for end stage renal diseases (ESRD) are dialysis and kidney transplantation, which has the major drawback of transplant rejection by the recipient's immune system [2–4] and the loss of homeostatic and endocrine functions of the kidney [5–7]. Consequently, the need for new and inexhaustible sources of kidneys is extremely urgent.

Recent advances in tissue engineering and regenerative medicine have shown promise for producing whole organs for transplantation. This concept involves the use of naturally occurring extracellular matrix (ECM) and stem cells or some adult cells. This type of natural scaffold is prepared by removing cellular components from the donor organs using detergents, a process known as decellularization [8]. Functional cells are then seeded on and/or within a natural scaffold, which could be referred to as recellularization. Researchers have applied this tissue engineering technology to produce functional substitutes for some organs, such as blood vessel [9–13], bladder [14], heart [15, 16], liver [17–19], intestine [20, 21], muscle [22], trachea [23–25], lung [26–28], and urethra [29].

Due to the complexity of the kidney, which includes over thirty different cell types, a vascular network and a large array of functional structures, a naturally occurring scaffold made of decellularized kidney ECM is a key step for a regenerative approach [30]. The renal scaffold must retain the three-dimensional structure of the organ, including vasculature, and the innate matrix components needed for promoting progenitor cell adhesion, migration, proliferation, and differentiation [31]. Several decellularization techniques have been applied to the kidney of some animals, such as rat [32], porcine [33, 34], and human [35]. However, these methods have many disadvantages, such as a complex process for producing the scaffold, insufficient evaluation, etc.

The goals of this study were to simplify the process for designing a renal scaffold in a rat model, evaluate the structure and function of the ECM, and produce a functional kidney by recellularizing these scaffolds. Our study shows that (1) rat kidneys can be decellularized to produce an acellular ECM scaffold through a simplified process; (2) these scaffolds maintain their structural and biochemical components as well as an intact vasculature that can be easily accessed. (3) the acellular scaffolds can provide a natural 3D structure for cell growth. (4) when implanted in rat recipients, the vascular network of the regenerated kidneys can sustain blood pressure and produce urine *in vivo*.

## RESULTS

### Decellularized kidney scaffolds retained structure and vascular network

**Figure 1:**
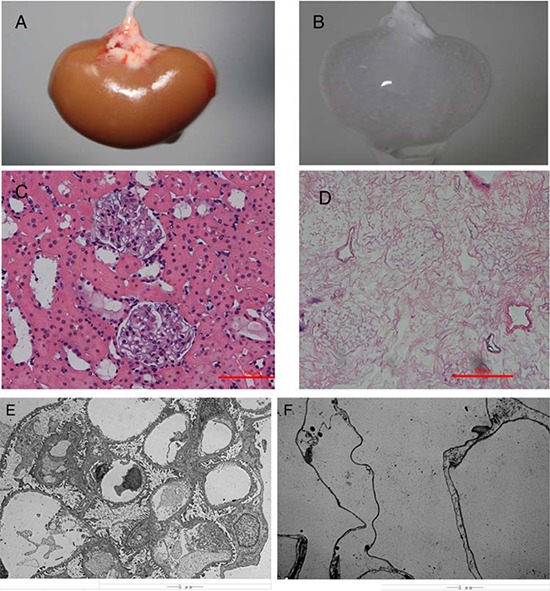
Decellularization of the rat kidney **A&B, Gross appearance of a kidney undergoing decellularization at the beginning A.** and end **B.** of the decellularization process. Hematoxylin and eosin staining of sections of renal cortex before **C.** and after **D.** SDS decellularization shows the absence of any cellular content and the integrity of the architecture of glomerular and tubular structures within the scaffold. Transmission electron microscopy images of decellularized kidney demonstrating the absence of cellular components and the preservation of basement membranes integrity. **E.** normal kidney; **F.** decellularized kidney scaffold.

Our simplified procedure for perfusion with SDS-contained buffer successfully decellularized rat kidneys as shown by a near transparent appearance (Fig. 1B). While hematoxylin and eosin staining showed pink staining typical for collagen, basophilic staining showed a lack of cellular nuclear material (Fig. 1D).

Electron microscopy indicated that no nuclear structure was found, however the integrity of the ECM was not disrupted in the acellular kidney scaffolds as shown by the presence of a continuous membrane of the Bowman's capsule, the basement membrane of the glomerular capillaries and mesangial matrix (Fig. 1E & 1F).

**Figure 2:**
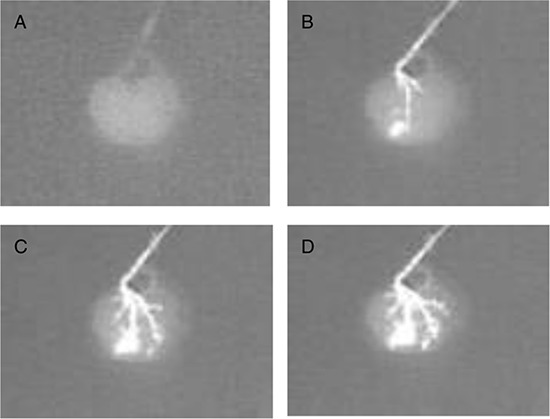
Fluoroangiography of a rat renal scaffold The fluoroangiography denotes the vascular tree is well represented through its hierarchical organization. The contrast medium was perfused at a rate of 0.5 ml/min. **A–D.** shows the image at times 0 s, 30 s, 60 s, 90 s.

We were able to retain an intact vascular network that retained hierarchical branching structures through the renal artery as depicted with contrast media (Fig. 2). In fact, the contrast media flowed progressively from larger vessels to smaller capillaries to eventually drain out through the stump of the renal vein. Importantly, the contrast media did not extravasate within the scaffold parenchyma.

### Decellularization process removes majority of DNA but preserves ECM and cytokines

**Figure 3:**
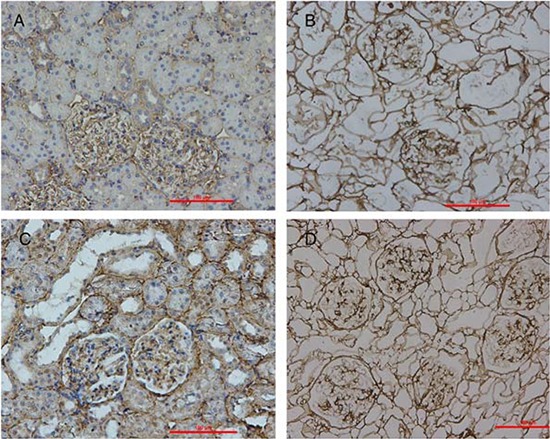
Localization of ECM proteins Immunohistochemical stains of cadaveric rat kidney sections (A&C) and decellularized scaffold (B&D) showing the distribution of collagen IV (A&B) and fibronectin (C&D).

**Figure 4:**
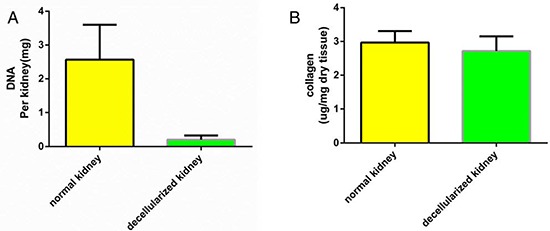
Quantitative assay of DNA and collagen content Biochemical quantification of DNA and total collagen in cadaveric and decellularized rat kidney tissue showing a reduction of DNA content and a preservation of collagen after perfusion decellularization. Data are shown as the mean ± s.d. Statistical significance ( *p* < 0.05) was determined by Student's *t* test.

**Figure 5:**
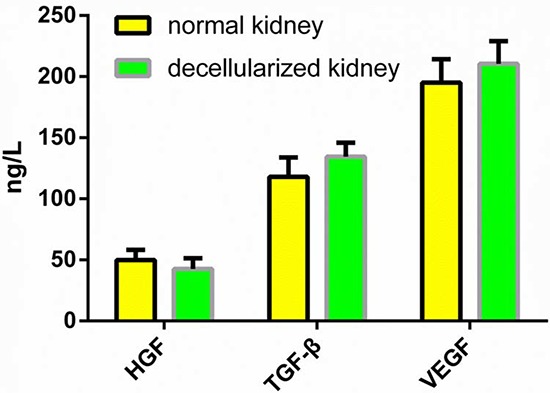
Quantitative assay of cytokines in DC kidney scaffolds The level of HGF, TGF-b and VEGF in the DC kidney is not different from that of native kidney. Data are shown as the mean ± s.d. Statistical significance ( *p* < 0.05) was determined by Student's *t* test.

The ECM of the decellularized renal scaffold, was preserved as seen by the distribution of specific ECM proteins, collagen IV and fibronectin, which were similar to that in native rat kidney tissue (Fig. 3A–3D). We removed approximately 95% of DNA in comparison to the native organ (Fig. 4A), while still retaining total collagen levels similar to those in cadaveric kidney tissue (Fig. 4B). Importantly, although the kidneys had been completely decellularized, ELISA assays showed levels of the cytokines HGF, TGF-b and VEGF in kidney scaffolds that were similar to that of the intact kidney (Fig. 5). These levels may be sufficient to contribute to renal regeneration.

### Seeded cells attach and proliferate on scaffold

**Figure 6:**
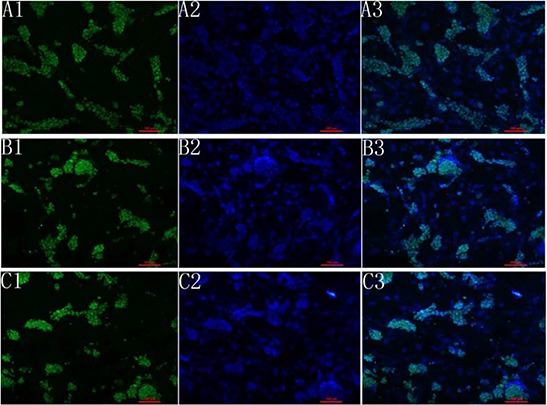
Fluorescence analysis of mES cells A1, B1, C1 shows the expression of OCT4, Nanog and sox2. A2, B2, C2 shows the dapi staining. A3, B3, C3 shows the merge picture.

**Figure 7:**
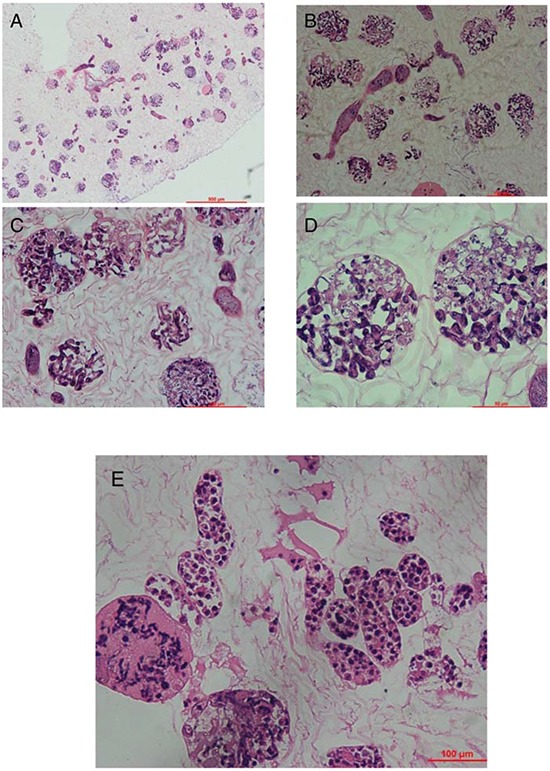
Recellularization of kidney scaffolds with mouse ES cells Hematoxylin and eosin staining of kidney scaffold seeded with mouse ES cells show homogeneous distribution of cells into glomerular, vascular structures, peritubular capillaries, and tubules. **A.** shows the gross appearance. **B–D.** shows the glomeruli part and **E.** shows the renal tubule.

We used immunohistochemistry to look for the expression of Oct4, Nanog and SOX-2 in cultured mouse vES cells (Fig. 6A–6F). These cells were able to attach and grow in the ECM after cell seeding and perfusion. Using hematoxylin and eosin staining, we showed that the cells were localized in vessels, gromeruli and renal tubules (Fig. 7A–7E).

### *In vivo* implantation of acellular scaffolds functioned normally for approximately two weeks

**Figure 8:**
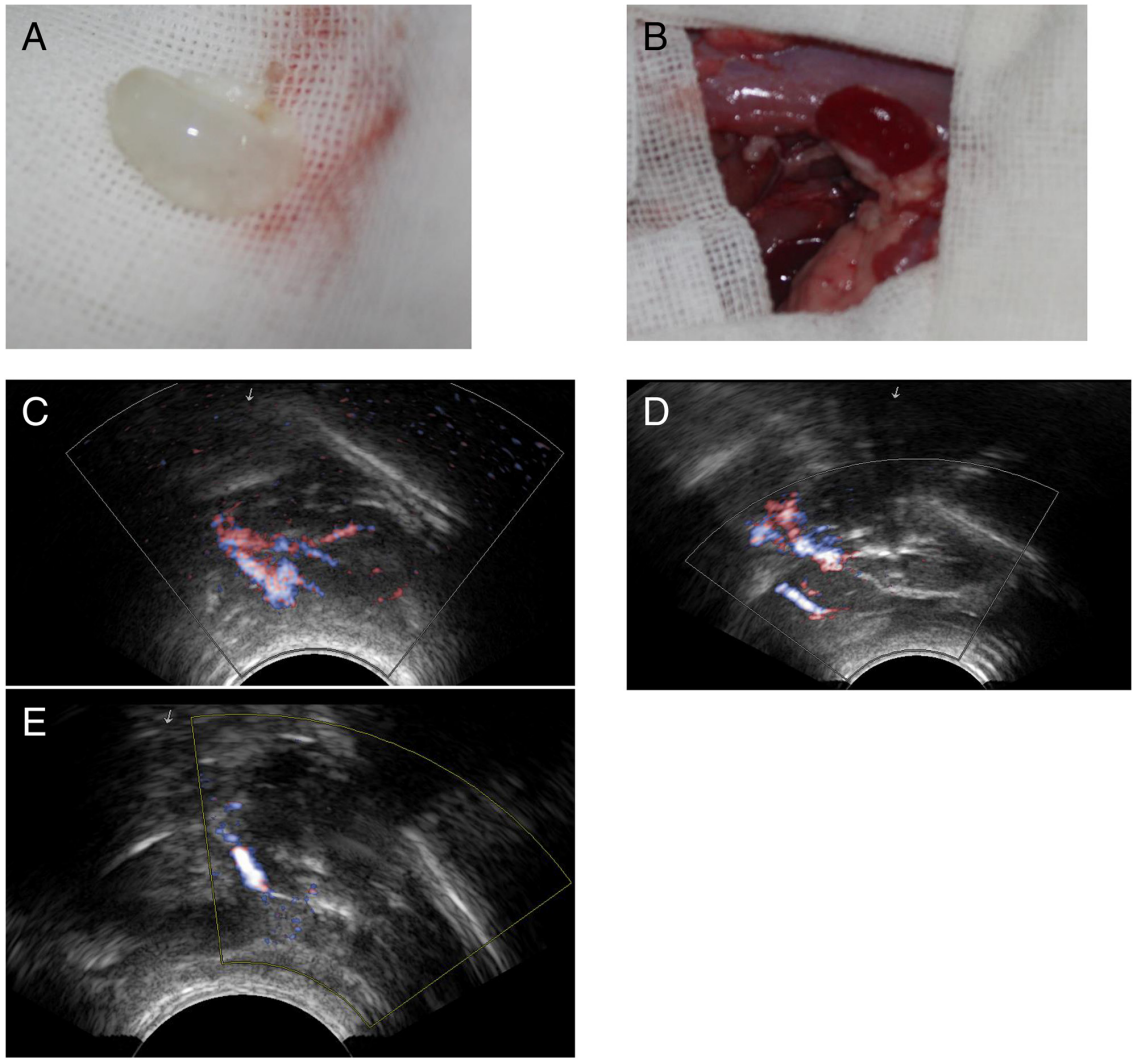
Implantation and explantation of the renal ECM scaffolds The gross appearance of the scaffold **A&B.** and the blood flow shown by B-scan ultrasonography **C–E.** (A), The acellular scaffold prior to implantation. (B), After removal of the clamps, blood flowed well within the whole scaffold. B-scan ultrasonography images of the implanted kidney scaffold obtained on day 2, weeks 1 and 2.

**Figure 9:**
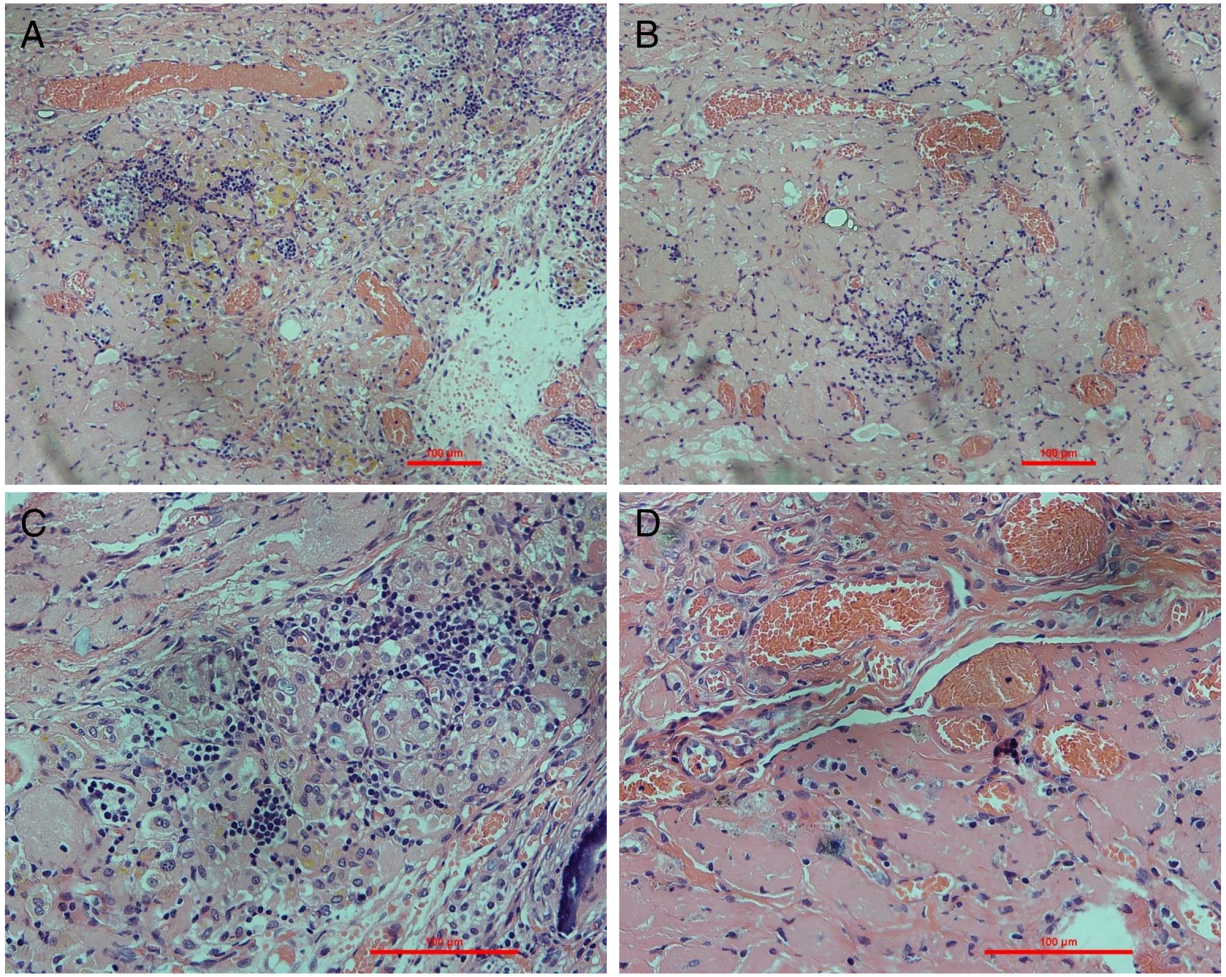
Histological examination of explanted renal ECM scaffolds Hematoxylin and eosin staining of the explanted scaffolds shows intense inflammatory cell infiltration. Glomeruli structures are well visible and show inflammatory cell infiltration. Some vascular structures are occluded by thrombosis or filled with red blood cells.

We were able to orthotopically implant rat renal scaffolds *in vivo* by reconnecting the vessels of the scaffold to the recipient's aorta and vena cava (Fig. 8A–8B). After we removed the clamps, blood flowed throughout the entire scaffold causing it to swell gently and acquire a color similar to that of normal kidneys. The scaffold implantation was well tolerated in animals and no adverse reactions were noted. No leaks were detected and no adverse events were recorded during surgery or follow up. We performed B-scan ultrasonography at day two,week one and week two post-surgery, showing that the blood flow disappeared on week two (Fig. 8C–8E). We removed the implanted scaffolds after two weeks. All the scaffolds were tightly adhered to surrounding tissues. However, as expected, the renal artery and the renal vein were obstructed by a massive thrombi, as there was no endothelium within the scaffold vasculature. Hematoxylin and eosin staining showed a massive, nonspecific inflammatory infiltrate and the vascular tree was completely obstructed by thrombi and trapped red blood cells (Fig. 9A–9D).

### *In vivo* implantation of regenerated kidneys show normal function

Throughout the entire test period, regenerated kidney grafts appeared well perfused without any evidence of bleeding from the vasculature. Regenerated kidneys produced urine shortly after removing the vascular clamps until the planned termination of the experiment.

**Figure 10:**
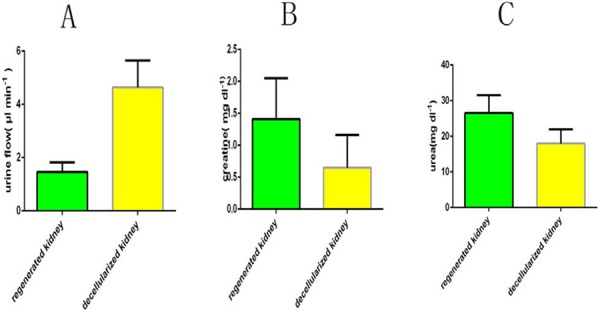
Functional test after transplantation of regenerated kidney Data are shown as the mean ± s.d (in three groups of data, *p* < 0.01). Statistical significance (*p* < 0.05) was determined by Student's *t* test.

Regenerated kidneys produced less urine than decellularized kidneys (1.8 ± 0.7 μl min − 1 (mean ± s.d.) compared to 4.9 ± 1.4 μl min − 1 in decellularized kidneys) with higher creatinine (1.4 ± 1.4 mg dl − 1) and urea (26.5 ± 1.6 mg dl − 1) than decellularized controls. (Fig. 10A–10C).

## DISCUSSION

Whole organ scaffolds containing intact vascular and ECM architecture hold great promise for the field of organ regeneration, and also bring new hope to patients who have end stage renal failure [18, 34–36]. To the best of our knowledge, this is the first study that assesses decellularized renal scaffolds completely. We achieved three goals: (1) to simplify the decellularization process and evaluate the decellularized scaffold from as many aspects as possible, (2) to provide sufficient evidence for kidney regeneration through the decellularization-recellularization technology; recellularize the renal scaffolds, leading to the formation of viable tissue; (3) to provide the recellularized kidney with some excretory function after orthotopic transplantation *in vivo*.

Methods of decellularization have varied between different research groups. We confirmed that detergent decellularization of whole rat kidneys is possible without the disruption of its ultrastructure [31, 32, 36]. We used the basic criteria, including (1) remove native cellular materials (DNA content at or below 50 ng DNA/mg tissue) [37]; (2) preserve native extracellular components and ultrastructure of the decellularized organ [17, 19, 38]; (3) preserve the intact vascular system.

The decellularized renal scaffolds we made retained vascular trees that were intact at all hierarchical levels. When orthotopically implanted in rats, the vascular tree could sustain a normal blood pressure. Residual SDS levels in the scaffolds were negligible. Overall renal ECM was also maintained, and some kinds of cytokines remained. The decellularized kidney scaffold was non-cytotoxic and promoted cell attachment and differentiation when seeded with ES cells.

Commonly reported decellularization protocols may take up to several hours, days or even weeks to remove all cellular materials [31, 32, 36, 39]. Such protocols do not guarantee preservation of the ECM micro-structure or crucial cytokines, which have been shown to be necessary for the generation of functional tissue constructs [40]. In contrast, our approach reduces the perfusion time but still preserves the micro-structure. SDS allows for rapid cell disruption and clearance of nuclear and cytoplasmic residues while triton X-100 takes effect slowly [41, 42] and may damage the collagen of the ECM [43]. We demonstrated that complete decellularization of rat kidneys can be safely achieved using SDS alone, as documented by the histologic and immunochemistry findings that confirmed complete cell removal without loss of structural ECM proteins and some kinds of cytokines. This criteria is important because residual DNA fragments in decellularized ECM lead to cytocompatibility issues *in vitro* and adverse immunological response upon implantation [44–46]. These results were matched with the research made by Bonandrini *et al*. [31], but our methods took much less time.

Another important consideration for organ decellularization is minimizing the undesirable alteration and loss of biologically active ECM components [47], This is important for direct cell seeding and the reconstitution of the cellular compartment and eventually for organ regeneration [48]. We were able to maintain key ECM proteins, including collagen IV, laminin, and fibronectin. The ECM proteins retained their location and their physiological organization after decellularization (Fig. 3). The ECM is often known for its role in maintaining the structure and three-dimensional shape of the respective tissue or organ. However, the ECM is actually in a state of dynamic reciprocity with the resident cell population [49, 50]. In this reciprocity process, some cytokines in the ECM, such as VEGF, TGF, etc., also play an important role in cell differentiation and growth. In our study, we demonstrated that although the rat kidneys had been completely decellularized, some cytokines still remained within the scaffolds in concentrations that might be sufficient to contribute to renal regeneration after recellularization. All these results were coincidedwith previous studies [36, 39].

An important criterion was the preservation of the vascular tree and the basic ultrastructure. Cells can only survive within an area less than 3 mm away from a source of nutrients and oxygen, a bioengineered body part cannot exceed such size to be viable [33]. So reconstruction of a complex kidney requires good preservation of a vascular tree at all hierarchical levels. In our study, the decellularized kidney showed a complete vascular tree and vascular imaging (Fig. 2B) We confirmed the hypothesis that full decellularization of kidney using this new protocol resulted in minimal to no damage to the native vascular architecture. Another proof of the functional integrity of the vascular tree was by the short-term observation after the implantation of the renal scaffold *in vivo*. Blood reperfused the kidney scaffold and we got a kidney-like macroscopic appearance. No bleeding or extravasation occurred in the post-operation period. Histologically, we confirmed the presence of the ultrastructure of gromeruli and renal tubules. Hematoxylin and eosin staining showed a massive nonspecific, inflammatory infiltrate completely obstructing the vascular tree with thrombi and trapped red blood cells. This was because there was no functional cell recellularization. To further test whether these decellularized kidneys could support cell attachment and growth, mouse ES cells were seeded into the kidney scaffold, and we performed recellularized kidney culture *in vitro*. Embryonic stem cells are available as established cell lines; they have the ability to grow indefinitely while maintaining pluripotency, and they have the potential to develop into and form any embryonic organ *in vivo* [52, 53]. In our study, the ES cells attached in to ECM scaffolds and grew well. These findings further confirm the integrity of the renal ECM scaffolds and that the cells and ECM interact with each other. The bioreactor system we made was well used for the recellularized kidneys. Our results provide evidence for the feasibility of accelerating whole kidney scaffold cell repopulation using controlled perfusion conditions.

Several groups are working on kidney regeneration by cell-scaffold techniques [31, 32, 34–36]. Song *et al*. reported that perfusion with endothelial cells and neonatal kidney cells to a decellularized kidney scaffold resulted in a recellularization of the kidney scaffold [36]. The techniques reported were good for kidney regeneration, but there remained some problems. It is unclear what the required time is for maturation of the seeded scaffolds. Also, the immuno-rejection problem should not be ignored. Stem cells are the ideal cell type for kidney regeneration. Ross *et al*. first reported similar research to engineer a recellularized kidney through ES cell repopulation [32]. This report was a milestone for kidney regeneration as it provides the possibility to recellularize kidney scaffolds with stem cells, but the methods for decellularization and recellularization were obsolete. The decellularization process was complicated and the amount of perfused cells that attached to the scaffold was much fewer than with our method. Bonandrini *et al*. shortened the decellularization process and recellularized the kidney scaffold with ES cells. However, the time for decellularization was much longer than that of our research and the cells that attached to the scaffold did not grow well. There was no functional test for the scaffold and recellularized kidney. These results suggest that the methods for complete kidney regeneration still need improvement. Some subtle alterations may lead to successful kidney regeneration.

There are some limitations to this study. First, the rodent model has, in itself, limitations of scale. We are also working on a porcine model [54]. Second, the time for culturation of the seeded scaffolds may be too short for maturation, possibly influencing the function of the recellularized kidney. Last, the techniques for recellularization should be improved to perfection.

In summary, this study provides a simplified, time-saving procedure for engineering decellularized scaffolds from rat kidneys that have had all cellular components removed while still preserving the ECM, vasculature, glomerular capillaries and tubular membrane.

We also could recellularize these decellularized kidney scaffolds and get a functional kidney. In this context, future studies can be focused on the methods of renal cell-seeding to improve the efficacy of recellularization and investigation of the mechanism that influences self-organization of cells into renal structures in porcine or human models.

## CONCLUSION

We developed an efficient and rapid protocol to produce a kidney scaffold from rat kidneys while preserving the vascular system and ECM. This provided an opportunity to demonstrate the feasibility of recellularizing the scaffolds with desired cells, including stem, renal and vascular cells. We confirmed that this is feasible with stem cells in a decellularized kidney scaffold. Overall, this study indicates that our ultimate goal of delivering a transplantable, immune-tissue free, engineered kidney to patients is possible.

## MATERIALS AND METHODS

### Kidney retrieval

We isolated rat kidneys for this experiments from male Wistar rats (Animal Center of Shandong University, Jinan, China) weighing 220–400 g. All animal procedures were approved by the animal ethics committee of Shandong University (Jinan, China) and followed the Guide for the Care and Use of Laboratory Animals published by the U.S. National Institutes of Health (NIH Publication No. 85–23, revised 1996). After anesthesia with isoflurane, a longitudinal abdominal incision was made and we transected the renal artery, vein and ureter and retrieved the kidney. Then we cannulated the renal artery with a 24-gauge cannula (XinHua Medical, China) and the ureter with a 26-gauge cannula (XinHua Medical, China).

### Preparation of decellularized kidney scaffolds

The kidney was placed in an *ad hoc* device. The cannula inserted into the renal artery was connected with an eristaltic pump (YX1515X-A; Baoding Longer Precision Pump Co., China) to allow continuous rinsing with various detergents. Solutions were perfused at an approximate rate of 2 ml/min in the following order: 0.01 M phosphate buffered saline (PBS, pH 7.4) for 15 min, 0.5% sodium lauryl sulfate (SDS) for 4 h, and then PBS for 24 h to remove SDS.

### Characterization of the decellularized scaffold

#### Histological examination

To assess cell and nuclear clearance as well as preservation of collagen, hematoxylin & eosin and Masson Trichrome staining (Solarbio, China) were performed after fixation in 10% formalin, paraffin embedding, and sectioning.

#### Ultrastructural observation

Transmission electron microscope was used to examine the extracellular matrix in the DC kidney scaffolds. DC kidney scaffolds were fixed with 2.5% (v/v) glutaraldehyde in 0.1 M sodium cacodylate buffer (pH 7.4) overnight at 4°C and post-fixed with 1% osmium tetroxide for 1 h at 37°C. The samples were then dehydrated with a series of acetone solutions with increasing concentrations, infiltrated with epon resin and baked overnight at 65°C. Ultrathin sections (80 nm) were prepared, stained with 2% uranyl acetate and lead citrate, and observed under a Hitachi electron microscope (H7500; Japan) at 70 kV. We recorded images with a gatan 830 high resolution CCD digital camera.

#### Immunohistochemistry for ECM

Tissue slides were fixed in 4% paraformaldeyhyde, quenched for endogenous peroxidase in 0.3% Hydrogen Peroxide, blocked with 4% normal goat serum in PBS and incubated with primary antibodies for 24 h at 4°C. They were incubated with biotinylated secondary antibodies for 30 min at room temperature. The following primary antibodies were used: mouse monoclonal anti-Fibronectin antibody (Abcam, Cambridge, MA, USA, ab194395,1:200), rabbit polyclonal Anti-Collagen IV antibody (Abcam, ab6586,1:200).

Enzyme-linked immunosorbentassay (ELISA) for quantitative analysis of cytokines in kidney scaffolds.

Total protein in DC kidney scaffolds or intact kidney was extracted using an ELISA kit (R&D Systems, USA). The concentrations of various cytokines including VEGF, TGF-β, HGF were assayed with a microplate reader at 450 nm.

#### Imaging of the vasculature

To confirm the integrity of the vascular tree and demonstrate that fluid injected into the vasculature flowed throughout rather than extravasated throughout the organ, x-ray fluoroscopy was performed on the bench before implantation using a Siemens SIREMOBIL Compact L C-arm. Conray (iothalamate meglumine) (Mallinckrodt Inc, St. Louis, MO) contrast agent was diluted at a ratio of 1:50 in distilled water and perfused through the vasculature at a rate of 0.2 mL/minute.

#### DNA quantitation

The DNA content of fresh and decellularized kidney was quantified using the tissue DNA isolation kit (PureLink Genomic DNA MiniKit, Invitrogen) according to the manufacturer's instructions. Briefly, the samples were digested overnight using Proteinase K and a digestion buffer. Upon removal of RNA, DNA samples were isolated by spin column-based nucleic acid purification and the extracts were characterized spectrophotometrically (NanoDrop 1000; Thermo Scientific). Optical densities at 260 nm and 280 nm were used to estimate the purity and yield of nucleic acids, which were quantified on the basis of 280 nm absorbance.

#### Collagen quantification

Soluble collagen was quantified using the Sircol Assay (Biocolor), as per the manufacturer's instructions. Lyophilized tissue samples were first subjected to acid-pepsin collagen extraction overnight at 4°C and then to overnight isolation and concentration. Assay was then performed as instructed. All concentrations were determined on the basis of a standard curve generated in parallel, and values were normalized to original tissue dry weight.

#### Fluorescence analysis of the mouse ES cells

Mouse embryonic stem (ES) cells were used for recellularization. Fluorescence analysis was performed to identify the property of stem cells. Briefly, cells were washed once with PBS, and fixed using 4% PFA in 1X PBS. After fixation, cells were blocked and permeabilized for 1 h at 37°C with 5% goat serum/1X PBS with 0.1% Triton X-100. Subsequently, cells were incubated with the indicated primary antibody overnight at 4°C. The cells were then washed three times with 1X PBS and incubated for 1 h at 37°C with the respective secondary antibodies. 4,6-Diamino-2-phenylindole (DAPI) was used for nuclear counterstaining. Cells were washed three times with 1 ×PBS before analysis. The primary antibodies included rabbit polyclonal to Nanog (Abcam, ab106465), rabbit polyclonal to SOX2 (Abcam, ab97959), rabbit polyclonal to OCT4 (Abcam, ab18976), rabbit monoclonal to pax-2 (Abcam, ab79389). The second antibody was FITC-conjugated anti-rabbit antibodies (Zhongshan Goldenbridge, China)

### Biocompatibility evaluation of the renal scaffold

*In vitro* biocompatibility was tested with mouse ES cells. After decellularization and sterilization, the scaffolds were seeded with 5 × 10^7^/ml diluted in 2 ml medium from the renal artery and ureter. Cells were allowed to attach overnight, after which perfusion culture resumed. Perfusion media was infused through sterile access ports (Millipore) to minimize the risk of contamination. Media was allowed to equilibrate with 5% CO_2_ and 95% room air before reaching the cannulated renal artery at 1 ml/min for 7 days.

### Orthotopic transplantation of acellular scaffold

To test the mechanical strength of the vascular network and investigate whether the intact acellular scaffold would sustain physiologic blood pressure, 10 scaffolds were orthotopically transplanted in rats matched for age and weight to the scaffold donors.

Rats were placed in a supine position. After a median laparotomy and systemic heparinization through the inferior cava vena, the left recipient renal artery, vein and ureter were identified, dissected circumferentially. The left kidney was taken out. Then the vessel and ureter of the scaffold and the recipient was secured with a 10–0 silk ligation. After reperfusion, the abdomen was closed.

### Orthotopic transplantation of regenerated kidneys

After recellularization, we performed orthotopic transplantation of regenerated kidneys just as the protocol above for transplantation of acellular scaffold. We anastomosed regenerated left kidneys to the recipient's renal artery and vein. The ureter was cannulated to collect urine samples and performed consecutive observation for one hour.
